# Computer-Aided Design of Fragment Mixtures for NMR-Based Screening

**DOI:** 10.1371/journal.pone.0058571

**Published:** 2013-03-13

**Authors:** Xavier Arroyo, Michael Goldflam, Miguel Feliz, Ignasi Belda, Ernest Giralt

**Affiliations:** 1 Design, Synthesis and Structure of Peptides and Proteins, Institute for Research in Biomedicine (IRB Barcelona), Barcelona, Spain; 2 Servicios Cientifico Tecnicos, Universitat de Barcelona, Barcelona, Spain; 3 Intelligent Pharma, Parc Científic de Barcelona, Barcelona, Spain; 4 Departament de Quımica Organica, Universitat de Barcelona, Barcelona, Spain; Università di Napoli Federico II, Italy

## Abstract

Fragment-based drug discovery is widely applied both in industrial and in academic screening programs. Several screening techniques rely on NMR to detect binding of a fragment to a target. NMR-based methods are among the most sensitive techniques and have the further advantage of yielding a low rate of false positives and negatives. However, NMR is intrinsically slower than other screening techniques; thus, to increase throughput in NMR-based screening, researchers often assay mixtures of fragments, rather than single fragments. Herein we present a fast and straightforward computer-aided method to design mixtures of fragments taken from a library that have minimized NMR signal overlap. This approach enables direct identification of one or several active fragments without the need for deconvolution. Our approach entails encoding of NMR spectra into a computer-readable format that we call a *fingerprint*, and minimizing the global signal overlap through a Monte Carlo algorithm. The scoring function used favors a homogenous distribution of the global signal overlap. The method does not require additional experimental work: the only data required are NMR spectra, which are generally recorded for each compound as a quality control measure before its insertion into the library.

## Introduction

Fragment-based drug discovery has emerged in the past decade as a powerful tool for drug development and is now widely applied both in academic and in industrial screening programs. Its success derives from the structural simplicity and relatively low molecular weight (150 to 300 u) of the fragments, which contrast with the more complex, medium-weight compounds normally employed in high-throughput screening (HTS). Using fragments has three main advantages over using larger compounds: firstly, the chemical space is significantly smaller, and therefore, can be explored more efficiently; secondly, the hit rates are 10 to 1000 times higher; and lastly, fragments often show high ligand efficiency, thereby facilitating work to improve their affinity [1].

Various biophysical techniques such as X-ray crystallography, surface plasmon resonance and NMR have been exploited for fragment screening, where they must provide reliable detection of the mostly weak interactions between fragments and the target, with a low occurrence of false positives and negatives.

NMR-based fragment screening methodologies have become very popular, as they fulfill these requirements excellently. The only disadvantage of NMR compared to other screening methods is its low intrinsic sensitivity. To compensate it, and to increase throughput, researchers often assay fragment mixtures, rather than single fragments, in NMR-based screening [2]–[3]. Modern NMR-based screening methodologies rely mainly on ligand observation experiments in which either a conventional NMR parameter of the ligand (*e.g.* relaxation properties), or the intermolecular proton magnetization transfer from the protein to the ligand, is evaluated [3]. In theory, both types of experiments enable direct identification of one or several binding fragments in a mixture that also contains non-binding fragments, yet they do not require deconvolution. The only requirement is that the NMR signals of the fragments in the mixture can be distinguished one from another, so that they can be evaluated separately, a subject which is only marginally covered in the literature [4]–[5].

We addressed the NMR signal overlap issue through the following process: firstly, conversion of NMR data for compounds from our in-house fragment library into a meaningful, computer-readable format; secondly, evaluation of different computational algorithms for the task of reducing signal overlap; thirdly, preparation of *in silico* mixtures of fragments taken from our in-house library, based on zero or near-zero signal overlap; next, confirmation that the *in silico* mixtures behave similarly to real (chemically synthesized) fragment mixtures; and finally, testing of the general adaptability of the algorithm.

## Methods

### NMR Spectrometry and Computation

All NMR spectra were acquired on a Varian Inova 500 MHz spectrometer with a 5 mm PFG Penta Probe at 37°C. S/N for ^1^H was 815∶1 (0.1% ethylbenzene in CDCl_3_). All calculations were performed on an SGI® Altix® 4700 server (64 cores, 128 GB RAM).

### Sample Preparation, and Generation of Fragment *Fingerprints*

Stock solutions (100 mM in 9∶1 DMSO-d_6_/D_2_O) of each compound from our in-house fragment library were prepared, and then inspected visually to confirm solubility. Soluble compounds were further diluted to 1 mM in deuterated buffer (25 mM phosphate, 50 mM NaCl, 11 µM *t*-butanol, pH 7.0), and their individual 1D-^1^H-NMR spectra were recorded using presaturation for water suppression. The purity and identity of each fragment was manually checked in each spectrum. Compound concentration was calculated based on an internal standard (*t*-butanol). NMR data for all fragments that passed quality control were then translated into computer-readable files, called *fingerprints*, by an in-house modified Varian script for automatic processing. The routine for signal integration was modified to integrate a narrow zone around each signal and to create an ASCII file for each NMR spectrum, consisting of the integration range of each signal and the value of the integral. Therefore, the generated file comprises several regions defined by start and end values that mark the spectral regions containing signals. Raw data were adjusted for subsequent calculations by removing regions originating from H_2_O (4.780–4.530 ppm), DMSO (2.754–2.613 ppm) and *t*-butanol (1.320–1.130 ppm) and by reducing the size of all remaining regions by 50%. The library was analyzed, obtaining and average number of integration zones of 7.5±1.9, an average size of the integrations zones of 0.152±0.048 ppm, an average number of peaks of 12.7±6.7 and an average line width of the peaks of 3.2±1.5 Hz.

### Algorithms

The task of designing mixtures of a non-redundant pool of fragments is a case of the *knapsack problem*, one of the typical, non-deterministic polynomial time (NP-complete) problems widely described in the literature [6]–[7]. To solve this problem,four different types of algorithms grouped in two types were tested: deterministic (greedy [6]–[7] and backtracking [6]–[7]) and stochastic (Simulated Annealing (SA)and genetic [9]–[10]).

As starting point for all algorithms, we implemented the same data structure. This structure was an array with the compounds, each one with its respective fragments, where in deterministic algorithms, the solution array was progressively filled, while stocastics was initialized with a random permutation of the fragments.

We used both the greedy and the backtracking algorithms to maximize the number of library fragments that could be used for five-fragment mixtures that would not have any signal overlap. This was accomplished through a scoring function that maximizes the number of fragments (Table 1, A1 and A2). In this case, the valid criterion for extending partial solutions was the success of adding new fragments into the mixture, such that once the process arrives at a solution, no more fragments can be added. To facilitate searching in the greedy algorithm, the fingerprint library was sorted by spectral area coverage at the beginning of the procedure. For the backtracking algorithm, to amplify the screening in the solution space and shorten the time required, it was executed several times in parallel, using random starting points.

**Table 1:** Characteristics and performance of the tested algorithms.

Algorithm	Algorithm Type	Execution Time	Scalable	Optimization
**Greedy**	**Deterministic**	Very short	Yes	Very low
**Backtracking**	**Deterministic**	Very long	No	Low
**Genetic Algorithm**	**Stochastic**	Short	Yes	Not possible
**Simulated Annealing**	**Stochastic**	Short	Yes	High

The scoring function in the deterministic algorithms is based on achieving zero signal overlap (*Scoring = Ni*, where N_i_: number of fragments in the mixture) while in stochastic algorithms the scoring function is based on achieving minimal signal overlap (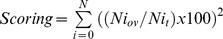, where N_ov_: number of overlapped signals of compound *i*, and N_t_: total number of signals of compound *i*).

In the SA and genetic algorithms, a different scoring function was used (Table 1, B1 and B2) to minimize overlap in each mixture. In the genetic algorithm, an initial population of 1000 candidates was established with standard conditions of selection, breeding and mutation taxes [9]–[10]. The evolutionary process was extended over 50 generations. We carried out 100 iterations of the SA algorithm in parallel with two million cycles, where the temperature value was close to zero. To study the scalability of the virtual libraries, the SA algorithm was then run 100 times independently, using the same algorithm conditions with the different lists. Finally, the effect of temperature was tested using values from 0 to 25000 in a virtual library of 500 fingerprints that contained an equal proportion of aliphatic and aromatic peaks.

### Generating Virtual Fingerprint Libraries

We modeled *virtual fingerprints* using parametrized values to mimic the fragment fingerprints obtained experimentally from our in-house library. A virtual fingerprint comprises a series of start and end points delimiting diverse peak regions. The number of peaks per fragment, and the position and width of each peak (integration range), are randomly established according to a Gaussian function. In this procedure, a Box-Muller transform is used to generate standard, normally distributed random numbers: *N(μ,σ^2^)*. Different mean values and variations were selected in each case (all in ppm). The number of peaks and the integration range follow a normal distribution of *N(8,4)* and *N(0.1,0.03)*, respectively. The peak position was determined using two combined normals [*N(2,1)* and *N(7.5,1.5)*] with different probabilities, producing three different distributions (strongly aliphatic, strongly aromatic and balanced). The effect of the library size on the solution was tested using four different sizes: 500, 1000, 3000 and 5000 fragments. The effect of the number of fragments per mixture was also assessed for each distribution-size combination: 5, 8, 10, 15 and 20 fragments.

## Results and Discussion

### Translating NMR Data into Fingerprints

All computational projects demand careful preparation of the input data, whose quality dictate the quality of the results. Therefore, the first issue we tackled in this project was to translate NMR spectra into a meaningful, computer-readable format. We chose to directly use the NMR spectra that had already been generated in the setup and quality control of our in-house library in order to avoid the necessity of performing any additional experimental work. The in-house script process and integrates narrow regions around each signal creating an ASCII file for each spectrum. This file comprises a collection of segments defined by the starting and ending chemical shifts of each integration zone. Thus, each fragment’s spectrum is an ensemble of different segments of signals and surrounding space. We refer to the entire ensemble as a *fingerprint*.

**Figure 1:**
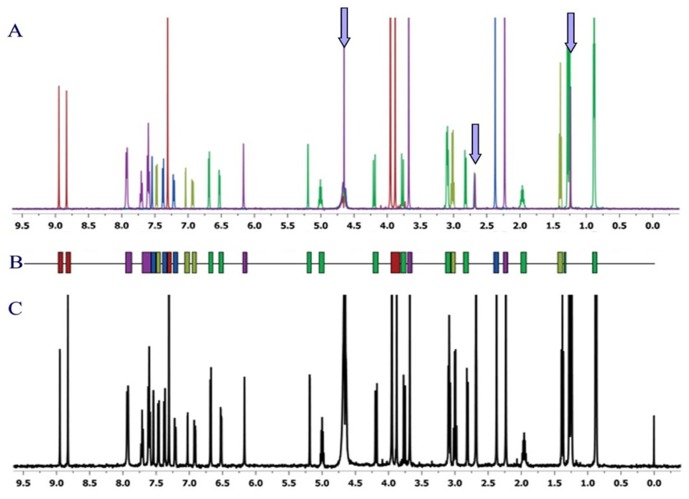
^1^H- NMR spectra sample. **A:** Overlaid ^1^H-NMR spectra of five different fragments (1 mM in sample buffer: 50 µM phosphate buffer pH 7.0, 50 µm NaCl, 3% DMSO-d_6_), recorded at 37°C and 500 MHz. The arrows indicate residual peaks from H_2_O, DMSO and *t*-butanol (internal standard). **B:** Fingerprint of an *in silico*-designed mixture with zero or near-zero signal overlap. **C:** 1H-NMR spectrum of the five fragments mixed together (500 uM each) under identical experimental conditions as in 1A (the signal at 0 ppm corresponds to DSS).

The raw data were adjusted for the subsequent calculations in two steps: firstly, signal regions common to all the spectra were defined (*i.e.* signals from H_2_O, DMSO, and *t*-butanol [internal standard]), and all segments that overlapped with these regions were partially or completely removed; secondly, since the size of each segment generated by the script was larger than that required to clearly separate the signals from each other, the size of all segments was reduced by a 50%. In fact, the size of the necessary zones varied from 30% to 90% of the size of the zones defined by the script. The in-house script provided too wide regions of integration and after analyzed them we checked that reducing the regions to the 50%, the 90% of the peaks included in the region provided by the script was recovered obtaining a more suitable size of the region of integration. Figure 1A shows several overlaid ^1^H-NMR spectra corresponding to single fragments; Figure 1B shows the fingerprints of the corresponding compounds, used to design fragment mixtures that would show nearly zero signal overlap; and Figure 1C shows the actual ^1^H-NMR spectrum of the mixture of fragments studied in the same NMR tube.

### Algorithm Evaluation

As explained, we examined four different algorithms for preparing five-fragment mixtures that would have zero signal overlap. Among the deterministic methods, the greedy algorithm could group only the 60% of the fragments into mixtures of five without causing signal overlap; while when it grouped the remaining 40% into mixtures of five, they exhibited strong overlap. The backtracking algorithm showed a similar behavior, making greedy methods advantageous owing to its relative simplicity and speed. Backtracking is a refined brute force approach: it systematically searches for a solution to a problem among all available options. In our case it made the finding of a final solution nearly impossible, due to the fact that the number of compounds demanded long calculation times. However, as a partial solution, the backtracking algorithm was able to group 75% of the compounds into five-fragment mixtures without overlap. Analysis of the remaining 25% of compounds revealed that they comprise complex fragments that have many signals located in the crowded aliphatic and aromatic regions. In light of this result, we realized that we needed to define a new scoring function based on *minimal* overlap for all the fragments from the library, rather than zero overlap for only some of these fragments. Another problem highlighted by the backtracking algorithm is that the required calculation time grows exponentially with the size of the data set. Therefore, we decided to explore stochastic algorithms, which we expected to be much faster and enabling the performance of multiple calculations in parallel. Genetic algorithms failed to provide a coherent solution, due to problems in the breeding and mutation steps, causing a loss of compounds in each evolutionary cycle. The problem showed by the genetic algorithm was that the fragments were stored in arrays, and the array was considered as an individual of the algorithm. Thus, when crossover of the best individuals were carried out, it was possible that some fragments were repeated in the same individual. Although in the formula was introduced a parameter in order to maximize diversity, it was not possible to totally avoid the fact that the algorithm remove those fragments to reach a global minima. Considering that the main interest was to keep all the fragments, the optimization with the genetic algorithm was considered as not possible. Contrariwise, the SA algorithm (Monte Carlo-Metropolis) yielded good results in a reasonable time. It can be run with libraries of up to 1000 compounds in less than 5 minutes, thereby enabling parallelization of massive independent runs. Unexpectedly, we found that temperature had a negligible effect on the results, as we describe later.

### Optimization of Mixtures from the In-house Fragment Library

**Figure 2:**
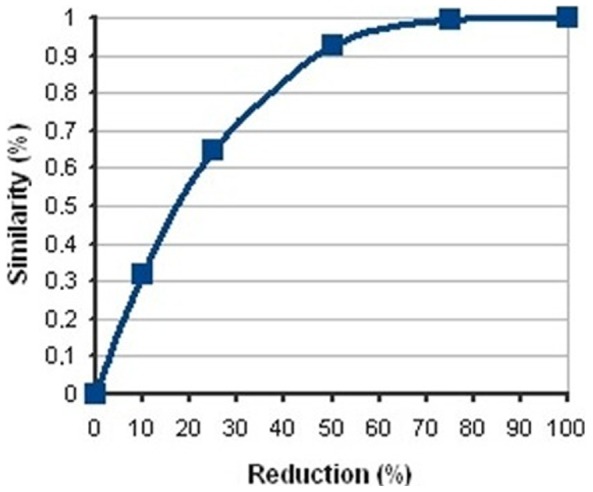
Effect of the reduction of the size of the fragments. X-axis showed %reduction of the size of all segments and y-axis %similarity between the number of peaks in each segment before and after the reduction.

We performed 100 independent runs of SA for the 342 fragments from our in-house library that passed quality control. One hundred random solutions were calculated in parallel by clustering the fragments into mixtures having the same number of fragments. Based on the assumption that each component contributes equally to the final score of the mixture, the random mixtures showed an average signal overlap of 44% per compound. After optimization by SA, the average global signal overlap per compound was reduced to only 2% (an improvement of a 42%). The *in silico* results were confirmed by mixing the appropriate fragments into mixtures who’s ^1^H-NMR spectra were then recorded. A peak list was generated for each mixture and compared to the fingerprint of each fragment. After the 50% reduction in segment size, more than 92% of the peaks matched with the regions corresponding to the fingerprints (Figure 2). Based on these results, we concluded that the *in silico* fragment mixtures corresponded to the real ones.

### Evaluating Algorithm Adaptability with Virtual Libraries

**Figure 3:**
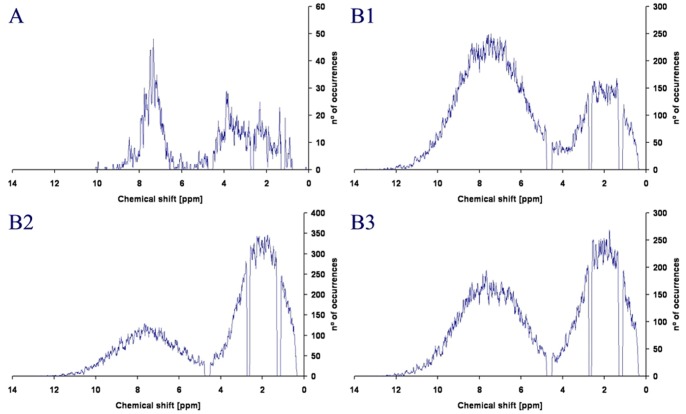
Histogram representation of the 1H-NMR peak distribution. A: 1H-NMR peak distribution of the 342 fragments of the in-house library. **B(1–3):** Analogous plots for the virtual libraries of 3000 fragments having the following signal density distributions: 50% aromatic, 50% aliphatic (**B1**); 70% aromatic, 30% aliphatic (**B2**); and 30% aromatic, 70% aliphatic (**B3**).

**Figure 4:**
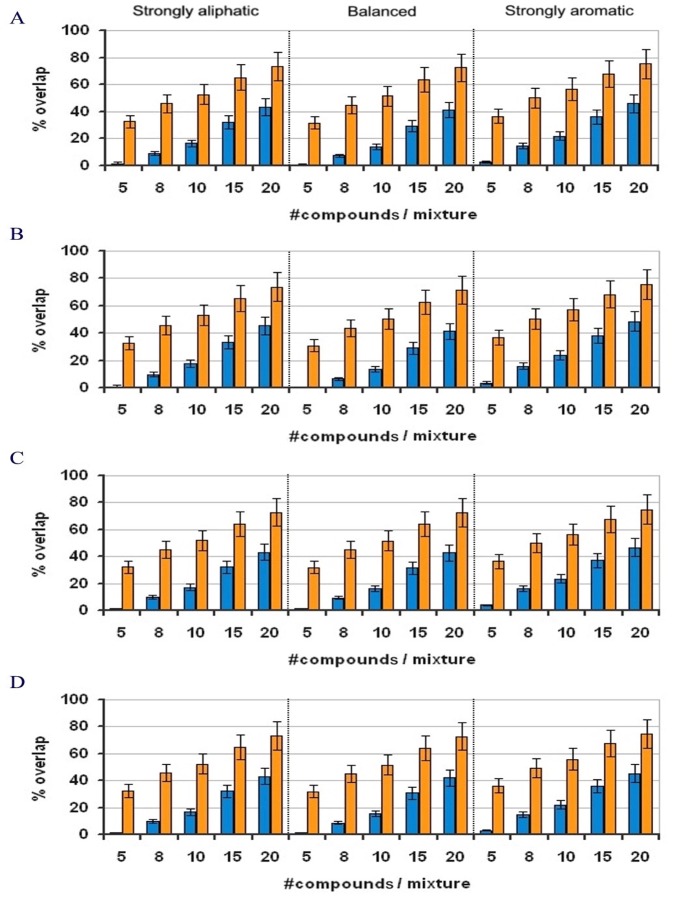
Results for the virtual library of 3000 fragments comparing randomly constructed (orange) and optimized (blue) libraries. The parameters and values tested were: *library size* (A-D: 500, 1000, 3000 and 5000, respectively); *peak distribution* (strongly aliphatic, strongly aromatic and balanced); and *number of compounds per mixture* (5, 8, 10, 15 and 20). For each set of conditions, the SA algorithm was run one hundred times independently.

Given the size of our in-house library, we were unable to study scalability and other variables that could affect the SA algorithm. Therefore, we designed a virtual fingerprint-generator to produce *virtual libraries*. A total of twelve different libraries, differing in global size and peak distribution, were generated. Library sizes of 500, 1000, 3000 and 5000 fragments were chosen. Three different distributions were selected, representing libraries whose fragments’ NMR signals were strongly aliphatic, strongly aromatic or balanced. Figure 3 shows a representative example from one of these virtual libraries. The twelve virtual libraries were then used to test the ability of the SA algorithm to reduce the global signal overlap (compared to that of a random solution) for mixtures of five to twenty fragments (Fig. 4).

For each set of library size, peak distribution, and mixture size, the SA algorithm was run independently 100 times, and the results were compared to those from random clustering of fragments into mixtures. Interestingly, library size and peak distribution had no significant impact on the SA algorithm while mixture size affected the global signal overlap for both the random and the optimized mixtures. Whereas the SA algorithm achieved a global signal overlap close to 0% for five-fragment mixtures, the overlap increased when increasing the mixture size. However, in the random mixtures, for each set of conditions, the SA algorithm still reduced signal overlap by 35%, regardless of the mixture size.

For mixtures of 20 fragments, SA reduced the signal overlap to the level corresponding to a randomly assembled mixture of eight fragments. Although screening of mixtures containing more than eight compounds is not currently common practice, future improvements in NMR sensitivity may enable this for cases in which signal overlap is low enough that deconvolution is not required. Theoretically, one can expect that with bigger fragment libraries we should obtain a lower percentage of global overlap between fragments owing that the number of fragments that can be mixed is higher. However, *in-silico* results showed that the bigger the library the higher the difficulty of finding the best combination of fragments in the mixtures. This behavior is explained by the fact that in the second case, the probability of each fragment of being chosen by the SA algorithm is lower. Thus, it is more difficult to find the best combination, although Figure 4 shows that the level of overlap for each group of 500, 1000, 3000 and 5000 were nearly equivalent.

### Effect of Temperature on SA

**Figure 5:**
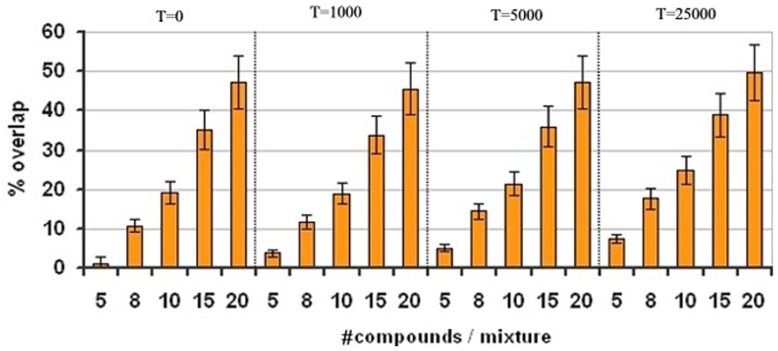
Effect of temperature on signal overlap. The experiment was performed with a virtual library of 500 compounds and a peak distribution of 50% aromatic and 50% aliphatic.

Temperature is normally an important variable in the SA algorithm: during optimization it controls the acceptance of uphill moves, thereby avoiding local minima. To determine the best temperature value for the algorithm, the impact of temperature on the capacity to reduce signal overlap was tested with equally distributed libraries of 500 fragments each. The temperature value ranged from 0 (no uphill moves allowed), to 25000 (nearly 100% of uphill moves allowed by using a value larger than a fully simultaneously overlapped score of two compounds). Surprisingly, modifying the temperature had no positive effect on the algorithm, independently of the conditions tested (Figure 5). It can be explained owing that each optimization step in the SA algorithm is not directly linked to the previous one, and consequently, each of these steps can have highly variable effects. Therefore, late uphill moves are translated into high increments in the scoring, making this effect nearly negligible owing that the algorithm reaches its minima much before that temperature has a real meaning.

### Conclusions

We have devised a powerful method for NMR screening of mixtures of fragments that entails translation of NMR spectra into *fingerprints*. Among the various algorithms evaluated to solve the problem of signal overlap, the SA algorithm offered the best optimization. As proof of concept, we used this algorithm to design five-fragment mixtures from our in-house library that showed an average signal overlap of only 2%.

We conceived virtual fragment libraries to evaluate the performance of the SA algorithm based on peak distribution (relative aliphatic or aromatic character of the library), scalability (*i.e.* library size), and temperature. Results suggest that the method is amenable to libraries of any size or nature. Furthermore, temperature had no effect on signal overlap.

This method could improve the efficiency of NMR-based fragment screening by simplifying detection of binding compounds, as it does not require any special computational hardware and, in the case of compounds whose NMR spectra are already available, it does not require any additional experimental work.
